# Pennsylvania State Examinations

**Published:** 1901-06

**Authors:** 


### PENNSYLVANIA STATE EXAMINATIONS.

THE annual meeting of the Pennsylvania State Board of Veterinary Medical Examiners for the examination of applicants desiring to obtain the license to practice veterinary medicine and surgery in the State of Pennsylvania will be held in Philadelphia, Pa., June 17th and 18th, at 9 A.M. each day. For any further information address the Secretary, W. Horace Hoskins, 3452 Ludlow Street, Philadelphia, Pa.

